# Genomic divergences among cattle, dog and human estimated from large-scale alignments of genomic sequences

**DOI:** 10.1186/1471-2164-7-140

**Published:** 2006-06-07

**Authors:** George E Liu, Lakshmi K Matukumalli, Tad S Sonstegard, Larry L Shade, Curtis P Van Tassell

**Affiliations:** 1 USDA, ARS, ANRI, Bovine Functional Genomics Laboratory, Beltsville Agricultural Research Center (BARC) – East, 10300 Baltimore Avenue, Beltsville, MD, 20705, USA; 2 Bioinformatics and Computational Biology, George Mason University, Manassas, VA 20110, USA

## Abstract

**Background:**

Approximately 11 Mb of finished high quality genomic sequences were sampled from cattle, dog and human to estimate genomic divergences and their regional variation among these lineages.

**Results:**

Optimal three-way multi-species global sequence alignments for 84 cattle clones or loci (each >50 kb of genomic sequence) were constructed using the human and dog genome assemblies as references. Genomic divergences and substitution rates were examined for each clone and for various sequence classes under different functional constraints. Analysis of these alignments revealed that the overall genomic divergences are relatively constant (0.32–0.37 change/site) for pairwise comparisons among cattle, dog and human; however substitution rates vary across genomic regions and among different sequence classes. A neutral mutation rate (2.0–2.2 × 10(-9) change/site/year) was derived from ancestral repetitive sequences, whereas the substitution rate in coding sequences (1.1 × 10(-9) change/site/year) was approximately half of the overall rate (1.9–2.0 × 10(-9) change/site/year). Relative rate tests also indicated that cattle have a significantly faster rate of substitution as compared to dog and that this difference is about 6%.

**Conclusion:**

This analysis provides a large-scale and unbiased assessment of genomic divergences and regional variation of substitution rates among cattle, dog and human. It is expected that these data will serve as a baseline for future mammalian molecular evolution studies.

## Background

Many mammalian species have long served our human society by providing food, materials, and labor, providing companionship as pets, and serving as model organisms for biological studies. Besides the seven mammals (human, mouse, rat, chimpanzee, macaque, dog and cattle) whose genomic sequence data are already available, 16 eutherian mammals have been proposed for low-coverage genome sequencing efforts [1]. Comparative genomics has been proven to be a powerful strategy to identify important evolutionary changes among these mammalian species [2]. Evolutionary changes, which have shaped the mammalian genomes, include both small-scale (point mutations, microsatellite slippage, insertions/deletions) as well as large-scale events (transpositions, genomic rearrangements and segmental duplications). Knowledge of mutation rates is critical for building evolutionary timescale, discovering conserved noncoding functional elements, identifying evolutionary processes like positive selection, and understanding heritable diseases [3].

Earlier studies on mammalian evolution were limited by the lack of large-scale genomic sequence data and were dependent upon PCR cross-amplification of limited numbers of mitochondrial and nuclear genes. Therefore, these sampled sequences were often limited to closely related species and had a bias towards conserved unique regions. This also resulted in repetitive sequences being excluded from genomic divergence calculations in these earlier studies. As the remnants of transposition events, repetitive sequences are one of the most predominant features of mammalian genomes (for example, 40–50% of the human genome are repeats) [4,5]. Repeats have been shown to play an important role in mammalian genome evolution [6,7]. Depending on their time of origin, repeats can be divided into ancestral repeats (AR: arrived before a speciation event, and thus shared by both species) and lineage-specific repeats (arrived after a speciation event). Recently it has been shown that virtually all ancient repeats evolve neutrally [8]. As one class of nonfunctional neutral sequences, ancient repeats have been used to estimate neutral mutation rates [9-11]. Several recent studies have indicated that neutral mutation rates (not substitution rates which are the combined effects of mutation and selection) in mammals have been relatively constant [12], except for the discrepant results from rodents, which were shown to mutate as much as 2-fold faster than other mammals [9,11].

With the availability of the human, mouse, rat and chimpanzee genome assemblies, whole genome-wide comparisons and analyses have been generated using primates and rodents (such as human vs. non-human primates, mouse, and rat) [4,5,9,13-15]. Targeted comparative sequencing efforts (the ENCODE – ENCyclopedia Of DNA Elements Project) also have generated megabases of high-quality genomic sequence for dozens of mammalian species [2,16,17]. Recent studies also have measured mutation rates [18], their regional variation [19,20] and their covariation with other genomic events in human, mouse and rat[11,21]. A local alignment algorithm, blastz [22], has been used to align human, mouse and rat genomes [9,14,21]. On the other hand, a global alignment algorithm, mlagan, has been used to generate multiple alignments in the "greater *CFTR *region" [23]. A comparison of results derived from local versus global alignment algorithms would be of interest.

With the dog draft assembly (July 2004, canFam1)[24], the cattle draft assembly (March 2005, bosTau2)[25] and cattle BAC library resources [26] now available, a large-scale genomic comparison was initiated to assess the nature and pattern of genomic variation among other mammalian orders; i.e. artiodactyls (Cattle, *Bos taurus*) and carnivores (Dog, *Canis familiaris*) as compared to primates (Human, *Homo sapiens*). To avoid any potential genome assembly artifacts, the project began with high-quality finished genomic sequences from cattle BAC clones, rather than the cattle draft assembly. The three-way multi-species global alignments (ranging in alignment length from 67 to 491 kb) were generated from the orthologous sequences of cattle, dog and human using an optimized global alignment algorithm to provide a platform for analyzing genomic variation. The lineage, which led to the last common ancestor (LCA) of cattle and dog, was estimated to have diverged from human approximately 92 million years ago (mya) followed by the estimated separation of cattle and dog 83 million years ago [27,28]. The overall objective of this study was to assess patterns of single-nucleotide mutations across genomic regions and among different sequence classes in the mammalian lineages.

## Results

### Orthologous sequences and alignment validation

A total of 84 ortholog trios were identified though a sequence similarity search, which included 10.5 Mb of cattle sequences, 9.3 Mb of dog sequences and 11.1 Mb of human sequences. The putative ortholog trios were further confirmed by reciprocal blast [29]. These ortholog trios were placed to all human chromosomes (chr) except for chr 9, 15, 19 and Y (see Additional file 1 Table S3).

Two strategies were implemented to align these orthologous sequences using the global alignment algorithm – mlagan: 1) optimizing the alignment parameters and 2) applying a post-alignment filter. In order to establish the optimal parameters to treat indels in global alignment, 5 random sets of pairwise sequence alignments were analyzed between cattle-dog, cattle-human and dog-human. Using the software lagan [23], a series of gap opening and extension penalties were tested for their impact on the frequency of single nucleotide and insertion/deletion events (see Additional file 1 Fig. S1). The following tests were performed to select the optimal alignment parameters that minimized sequence divergence and the number of indels. First, the natures of the sequences underlying insertion/deletions were analyzed. Alignment parameters (gap opening penalty of -1,000 and gap extension penalty of -10) were favored because insertion/deletions were effectively treated as a single event. Second, the overall estimates of sequence divergence (Table 1) were compared with earlier phylogenetic studies using conserved coding regions [12,30] or the greater *CFTR *region aligned by blastz [2]. The estimated overall sequence divergences in our analyses (cattle-dog: 0.3228 ± 0.0005, cattle-human: 0.3717 ± 0.0007, and dog-human: 0.3583 ± 0.0006 change/site) were generally comparable to previous studies [2,10,12,30]. Third, 73,728 randomly selected cattle BAC end sequences (BES) from CHORI-240 [31] were mapped onto the human genome assembly Build35 [32]. Similar results were observed when alignments of BAC end sequences were compared with our optimal global alignments. The variation distribution pattern of these BES alignments (400–500 bp) (G.E. Liu et al, unpublished results) was remarkably similar to the distribution observed for non-overlapping 500-bp windows generated from optimal global alignments (see Additional file 1 Fig. S5B).

Despite the optimization of alignment parameters, suboptimal or ectopic alignments occasionally occurred. Suboptimal alignments were defined as those alignments that exceeded 3 standard deviations of the mean pairwise K2 divergences in a sliding window analysis (See Methods), which were removed using a post-alignment filter. Although such suboptimal alignments composed less than 5% of aligned bases, these alignments were not considered in our analysis to avoid overestimation of genomic divergence.

**Figure 1:**
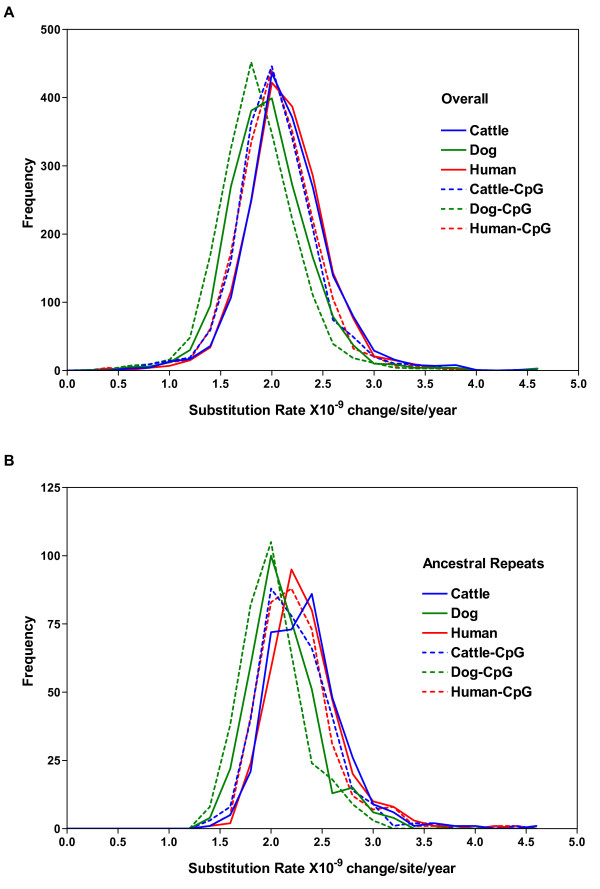
**Distributions of Substitution Rates in Cattle, Dog and Human**. (A) Histograms of the local substitution rates in aligned sequences (84 loci, 5.5 Mb aligned bases, 1,794 windows). (B) Histograms of the local substitution rates in aligned ancestral repeats (84 loci, 1.2 Mb aligned bases, 353 windows). All measures were computed in non-overlapping 3-kb sliding windows for cattle-dog-human multiple sequence alignments. These rates were calculated in multiple comparisons assuming branch times of the cattle, dog and human lineages from the LCA of cattle and dog of 83, 83 and 101 mya, respectively. Suboptimal alignments were excluded. The cattle branch: blue; the dog branch: green; and the human branch: red. The dashed lines were computed after removing CpG dinucleotides.

A total of 84 three-way multiple sequence alignments were generated with a combined alignment length of 15 Mb, consisting of 5.5 Mb of aligned bases and 1,794 non-overlapping windows of 3 kb (Fig. 1 and Additional file 1 Fig. S5A). The cattle-dog-human multiple alignment lengths ranged from 66,960 to 491,059 bp with a mean and standard deviation of 184,608 ± 79,744 bp. All individual alignments and patterns of single-nucleotide variation were manually inspected and are available online [33].

### Branch lengths in various sequence classes

Comparative genomic analyses were performed on these 84 three-way multi-species global alignments. The branch lengths and substitution rates of cattle, dog and human are shown in Table 1. The average overall branch lengths were 0.1681 ± 0.0003, 0.1547 ± 0.0003, and 0.2036 ± 0.0003 change/site for cattle, dog and human, respectively. Similar degrees of branch lengths were reported in previous studies [12,30]. The genomic divergence between cattle and dog was the smallest with a value of 0.3228 ± 0.0005 change/site. The dog-human evolutionary divergence was 0.3583 ± 0.0006 change/site, which was less than the cattle-human divergence of 0.3717 ± 0.0006 change/site. As expected, these results confirm that artiodactyls and carnivores are the closest relatives, with primates being the most distant. Mutations at CpG dinucleotides occur frequently due to spontaneous deamination of methylated cytosines [34]. To remove any variation caused by differences in levels of methylation, substitution rates were estimated after removing CpG dinucleotides (Overall-CG, Repetitive-CG). The overall branch lengths decreased 5.1% (cattle), 6.2% (dog) and 4.8% (human) after removing CpG dinucleotides from all sequences within alignments (Table 1, Overall-CG). Alignments were further sorted into four sequence classes based on NCBI RefSeq [35] and RepeatMasker coordinates using the software MaM [36]. The total 5.5 Mb aligned sequences included 133 kb, 115 kb, 4.0 Mb and 1.2 Mb aligned bases from coding, UTR, unique noncoding (i.e. not annotated), and repetitive regions, respectively. Coding regions of 193 well-annotated RefSeq genes excluded both 3' and 5' UTR. Branch lengths in coding regions (cattle 0.0644 ± 0.0010, dog 0.0647 ± 0.0010, and human 0.0595 ± 0.0009 change/site) were only half of the overall branch length, reflecting that they are under strong purifying selection. The branch lengths in UTR regions (cattle 0.1676 ± 0.0003, dog 0.1538 ± 0.0003, and human 0.2021 ± 0.0004 change/site) were significantly larger than the coding branch lengths (t-test, for each species p <0.0001). The branch lengths in unique noncoding portions (cattle 0.1676 ± 0.0003, dog 0.1538 ± 0.0003, and human 0.2021 ± 0.0004 change/site) were slightly less than the overall branch lengths. In contrast, the aligned repetitive portions possessed the longest branch lengths (cattle 0.1830 ± 0.0006, dog 0.1668 ± 0.0006, and human 0.2221 ± 0.0007 change/site). These branch lengths decreased 4.4% (cattle), 5.2% (dog) and 4.1% (human) when CpG dinucleotide sites were excluded, suggesting higher substitution rates of CpG sites (Table 1, Repetitive-CG). The differences were significant between the branch lengths in unique noncoding vs. repetitive portions before and after removing CpG dinucleotides from repetitive elements (one-way ANOVA, cattle P = 0.0006, dog P = 0.0116, and human P <0.0001) for all 83 autosomal alignments.

### Regional variation of substitution rates

Substitution rates were calculated from the LCA of cattle and dog assuming branch times of 83, 83 and 101 million years for cattle, dog and human lineages, respectively [27,28]. A dramatic variation of substitution rates was observed between and within chromosomes according to the human placement. Table S4 (see Additional file 1) summarizes the substitution rates of AR for each individual clone or locus on each chromosome. Chromosome X accumulated fewer substitutions than autosomal chromosomes (cattle 1.771 ± 0.045, dog 1.680 ± 0.043, and human 2.083 ± 0.049 × 10^-9 ^change/site/year), supporting the existence of a higher mutation rate in the male than in the female germline [34]. Among autosomal chromosomes, HSA10 (Human chromosome 10), showed higher substitution rates (cattle 2.372 ± 0.057, dog 2.417 ± 0.058, and human 2.583 ± 0.059 × 10^-9 ^change/site/year) compared to rates in chromosome 11 (cattle 2.151 ± 0.028, dog 1.916 ± 0.025, and human 2.022 ± 0.025 × 10^-9 ^change/site/year). Substitution rates for HSA10 and HSA16 were significantly higher, while those for HSA14, HSA12 and HSA7 were significantly lower when compared to the average substitution rates in repetitive regions (t-test, all P <0.0001, see Additional file 1 Table S4).

Similarly, substitution rates varied significantly among individual clones or loci within one chromosome (see Additional file 1 Fig. S2, Table S4). For example, contig 01.01 (mapped to HSA7:30,585,342-30,707,957 and CFA14:46,029,765-46,135,257) showed high substitution rates (cattle 2.371 ± 0.080, dog 1.788 ± 0.065, and human 2.020 ± 0.068 × 10^-9 ^change/site/year), while contig 33.39 (mapped to HSA7:114,308,522-114,473,710 and CFA14:56,758,901-56,922,307) demonstrated low substitution rates (cattle 1.998 ± 0.081, dog 2.092 ± 0.083, and human 2.264 ± 0.086 × 10^-9 ^change/site/year), even though both belonged to the same chromosomes (HSA7 and CFA14).

Histograms of substitution rates in non-overlapping 3-kb sliding windows for overall (A) and repetitive (B) sequences (with and without CpG sites) are shown in Fig. 1. ANOVA tests were performed on variation in branch lengths of 3-kb nonoverlapping windows between and within autosomal chromosomes for each species. These included 6 types of sequences: Overall, Overall-CG, Unique noncoding, Unique noncoding-CG, Repetitive, and Repetitive-CG. The overall sequence comprised 83 autosomal alignments containing 1761 windows; the unique noncoding regions comprised 83 autosomal alignments containing 1290 windows; and the repetitive regions comprised 83 autosomal alignments containing 347 windows. All tests were statistically significant at P <0.0001.

**Figure 2:**
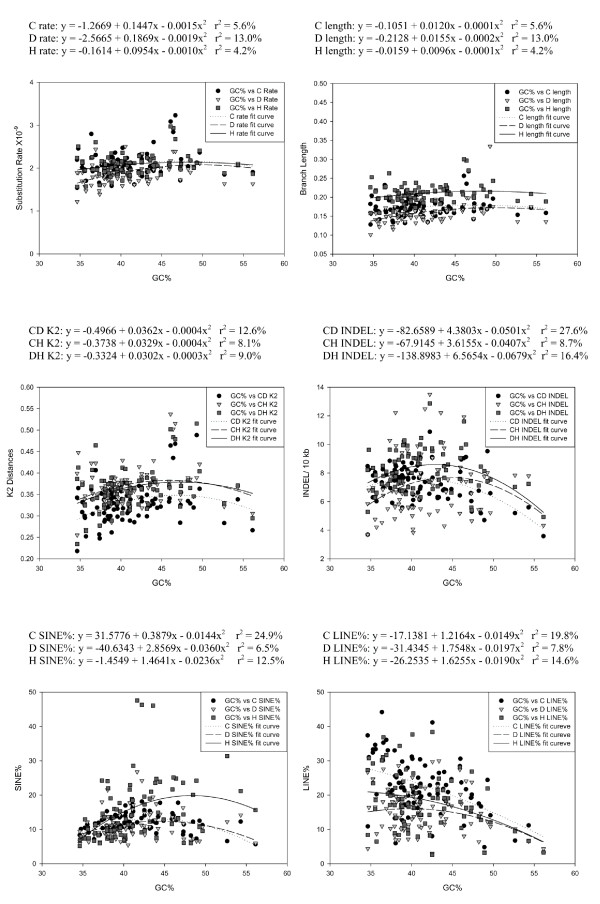
**Scatter Plots and Quadratic Fits on Average GC% for Substitution Rate, Branch Length, K2 Distances, INDEL/10 kb, SINE% and LINE%**. Scatter plots of substitution rate, branch length, K2 distance, INDEL/10 kb, SINE% and LINE% against average GC% in three-way alignments among cattle (C), dog (D), and human (H). Substitution rates (the top left panel) and branch lengths (the top right panel) were estimated for each species by the PAML package (Methods). For each pairwise comparison in three-way alignments, K2 distances (the middle left panel) and large indel frequency (>100 bp insertion/deletion event count per 10 kb, the middle right panel) were calculated. Other sequence properties in each species such as SINE% (the bottom left panel), LINE% (the bottom right panel) were also plotted. Quadratic fit curves are derived on each plot and their formulas are provided on the top of each panel.

The relationships of overall substitution rate, branch length, K2 divergence, indel rate per 10 kb, SINE% and LINE% on GC% were complex and were best fit by a quadratic function [9,11,21,37] (Fig. 2). It is worth noting that branch lengths (i.e. substitution rates after normalized by the divergence times) were well correlated among species – almost as well as the GC% distribution (see Additional file 1 Fig. S2), although branch lengths and substitution rates did not seem to correlate with GC% (Fig. 2). A positive coefficient for GC% but a negative coefficient for the square of GC% was obtained in all quadratic fit functions. The K2 divergences tended to increase over the GC% interval below 45%, whereas the plots tended to decrease above a GC% of 45%. However, all substitution rate and branch length fitting curves were relatively flat. This is consistent with an earlier observation of the discrepancy of rate estimation by the simple parametric model vs. the complicated rate model and maximum likelihood method at the high GC% isochores [38]. The quadratic fits for substitution rate on GC% had r^2 ^values of 5.6%, 13.0% and 4.2% for cattle, dog, and human, respectively. The quadratic fits for K2 divergence on GC% had r^2 ^values of 12.6%, 8.1% and 9.0% for cattle-dog, cattle-human and dog-human comparisons, respectively. Correspondingly, the quadratic fits for indel on GC% had r^2 ^values of 19.8%, 7.8% and 14.6%, respectively. The dramatic differences between SINE and LINE distribution relative to GC% agreed with the previous observations of their differential insertion bias and retention behaviors [4,9,14].

Loci with lower overall divergences were inspected for the presence of underlying RefSeq genes. As expected, many protein coding genes were under functional constraints. These constraints such as those on the *FOXP2*, *MET *and *SCAP2 *genes within the great *CFTR *region may explain the low overall divergences observed within that part of HSA7 [2]. When loci with high overall divergences were examined, it is interesting to note that a few protein coding genes were also detected. These included *CSMD2 *[39] (contig 38.45, HSA1:33,820,824-33,883,038), *FDFT1 *[40,41] and *CTSB *(contig 03.03, HSA8:11698086-11762835) [40,42,43]. These loci retained higher substitution rates even if only the AR regions were considered (see Additional file 1 TableS4).

### The differences of substitution rates between cattle and dog

The overall substitution rates were estimated to be 2.026 ± 0.003, 1.864 ± 0.003, and 2.016 ± 0.003 × 10^-9 ^change/site/year for cattle, dog and human, respectively (Table 1). Indeed, estimates of neutral mutation rates using ancient repeats (cattle 2.205 ± 0.007, dog 2.010 ± 0.007, and human 2.199 ± 0.007 × 10^-9 ^change/site/year) were comparable to previous studies (2.1–3.7 × 10^-9 ^change/site/year) [11], agreeing almost perfectly with the estimates from the human-mouse comparisons (i.e. 2.2 × 10^-9 ^and 4.5 × 10^-9 ^change/site/year in the human and mouse lineages) [9]. In all cases in Fig. 1 (Overall, Overall-CG, repetitive and repetitive-CG), the distributions of dog substitution rates (green) were shifted slightly to the left of those of cattle rates (blue), consistent with the faster rate of substitution in the cattle branch compared with the dog branch.

Relative rate tests were performed on a single merged alignment and on each of the 84 multiple alignments using Tajima's method [44,45]. Differences in mutation counts were assessed using the χ^2 ^test based on the assumption that mutation would not show a species preference. When using human as an outgroup, cattle had faster rates of substitution as compared to dog. Although the difference was relatively small (6%), it was significant by the χ^2 ^test (P <0.0001) when the merged alignment was tested. Almost two-thirds (54 out of 84) of the individual alignment rate tests supported that cattle had faster rates, while 11 of these rate tests supported that dog had faster rates (including 5 from the greater *CFTR *region). The remaining 19 out of 84 tests supported the molecular clock hypothesis for the cattle and dog lineages (including 3 from the greater *CFTR *region).

## Discussion

One of the fundamental challenges in large-scale comparative genomic analysis is to build biologically meaningful multiple sequence alignments [18,46]. A variety of biological events are known to create insertion/deletions including lineage-specific amplification of tandem repeats, homology-mediated genomic deletions and transposition events [34]. Local alignment algorithms, combined with the removal and reinsertion strategy of repeat elements, have been shown to reduce the number of gaps in DNA alignments and increase sensitivity [22,47]. This is particularly important for aligning the species like rodents which have high genome-wide substitution rates. However, the aligned ancient repeats may be enriched for those in more slowly changing regions, while the fast changing repeats may be too divergent for detection and alignment [21]. On the other hand, global alignment algorithms seem appropriate for species with low substitution rates like cattle, dog and human. Comparative gene mapping and chromosome painting studies have indicated that a remarkably slow rate of chromosomal change exists within several mammalian orders. Artiodactyls and carnivores are more conserved relative to humans than rodents [48-53]. In terms of genomic divergence, previous data [2] also suggests that cattle and dog are more conserved relative to human. But global alignment algorithms assume colinearity between sequences and do not specifically handle synteny breaking events like transpositions, rearrangements (such as microinversions) or duplications [54]. For example, global alignment algorithms may be ineffective to treat lineage-specific repeats which are closely matched such as young SINEs and LINEs, creating suboptimal alignments [21]. These suboptimal alignments may lead to less accurate estimates of sequence divergence. Therefore, in this study, alignment parameters were optimized and a post-alignment filter was applied to overcome the above limitation of the global alignment algorithm. The post-alignment filter effectively removed the suboptimal alignments from the mlagan output. Such suboptimal alignments appeared abnormal because they had extreme fluctuations in genomic divergences compared to their flanking sequences and were always associated with multiple gaps. Similar genomic divergence results obtained in the current study compared to earlier reports [10,12,30], confirm that our sequence datasets were representative and our alignment strategies were successful.

Our orthologous sequence datasets, comprised of 10.5 Mb of cattle sequences, 9.3 Mb of dog sequences and 11.1 Mb of human sequences, were placed on all human chromosomes except for chr 9, 15, 19 and Y (see Additional file 1 Table S3). As a control for sample bias and rate variation among these genomic regions, we mapped randomly selected cattle BAC end sequences onto the human genome assembly Build35 (73,728 BES from CHORI-240 [31]). A comparison of these BES alignments to our large-scale genomic alignments showed comparable results (G.E. Liu et al, unpublished results). Therefore, it is reasonable to believe that these datasets are sufficiently representative and robust to draw sound conclusions regarding rates and properties of mammalian genomic mutation.

However, our estimates were consistently larger than those in an earlier study of the greater *CFTR *region [2] and revealed significant rate differences between the cattle and dog lineages. Reanalysis of the alignments in that study (116 kb cattle, 122 kb dog, and 332 kb human sequences, 68 kb aligned bases) indicated that the dog-human divergence (0.3335 ± 0.0046 change/site) was significantly higher than the cattle-human divergence (0.3237 ± 0.0045 change/site) (Relative rate test, p <0.001). Comparable divergences were derived from our AR regions (369 kb cattle, 369 kb dog, and 485 kb human sequences, 157 kb aligned bases) from the same region (dog-human: 0.3856 ± 0.0035 change/site and cattle-human: 0.3870 ± 0.0035 change/site). In our study, no significant rate difference was detected between cattle and dog (Relative rate test, p = 0.251). One possible explanation is that the global alignment algorithm mlagan was used to create multiple alignments in the current study while pair-wise alignments were constructed by the local alignment algorithm – blastz in the earlier study. As discussed above, local alignment algorithms are known to be less efficient in identifying fast changing ancient repeats, which may be too divergent to detect and align. This could lead to the underestimation of the genomic divergences. On the other hand, use of a global alignment algorithm can recover the fast changing orthologous ancient repeats by taking into consideration the conservation of nearby unique flanking sequences. Discrepancies in the significance of rate variation between the small and large datasets also further highlight the importance of a large-scale sampling strategy.

As expected, different sequence classes were under different purifying selection pressures. Coding regions were under the strongest functional constraints with substitution rates at only half that of the overall substitution rates. It is interesting to note that substitution rates in unique noncoding portions were slightly less than overall substitution rates suggesting they may be under weak negative selection due to unidentified functional regions, regulatory domains, or unknown genes. Significantly higher substitution rates in repetitive elements before or after removing CpG dinucleotides indicate that CpG content is only partially the reason for high substitution rates. In addition, other factors like increased rates of gene conversion, relaxed purifying selection and unequal crossover among repeats may contribute to our observations.

The quadratic relationships between substitution rate, branch length, K2 divergence, indel rate per 10 kb and GC% were derived to explain regional variation. These results suggest that fluctuations in GC% predict an appreciable amount of the regional variation that was observed in mutation and indel rates, but leave the majority of the variation unexplained. Additional causes beyond GC%, including CpG content, recombination and other as of yet unknown factors are needed to explain the variation among mutation rates. Significant variation in mutation rates across genomic regions and among sequence classes strongly demonstrates that future studies of genomic variation should include multiple regions from different chromosomes. Another important observation is that regional variation in mutation rate is correlated among cattle, dog and human lineages over time. Regional correlations of mutation rates have been demonstrated and quantified genome-wide in human-chimpanzee, human-mouse and human-rat comparisons [9,14,20].

It is also interesting to note that a handful of protein coding genes were detected within a few cattle BAC clones with high neutral mutation rates. Several possible nonexclusive explanations for this phenomenon exist. For instance, the sequences compared may not have been orthologous. Within one gene family, paralogous genes could be confused with orthologous genes. Gene conversion may have occurred, which could considerably increase the genomic divergence [55]. In addition, high mutation rates or relaxed purifying selection could have occurred due to gene duplication [56,57]. These possibilities warrant further investigation. However, these rare events would not likely significantly change our estimates of mutation rates.

Measurement of the neutral mutation rate is crucial for validating molecular clock and neutral evolution theories [58,59]. The neutral mutation rate has been approximately estimated from neutral or close to neutral non-functional sites such as introns, pseudo-genes, unique noncoding intergenic regions, four-fold degenerate sites (4D sites) in coding regions (i.e. third codon position) and shared ancestral repeats. One way to identify regions under positive selection is to focus on DNA segments with significantly higher mutation rates [56]. Genomic regions that are changing significantly slower than the neutral rate because of purifying selection contain potentially conserved noncoding functional elements [11,21].

Estimates of the neutral mutation rates in this study, which are in agreement with many previous reports [2,12,30], show that mutation rates in the cattle and dog lineages are slower as compared with those in rodents. However, our estimates around 2.0–2.2 × 10^-9 ^change/site/year are in the lower end of the reported range (2.1–3.7 × 10^-9 ^change/site/year) [11]. These differences could result from the usage of 4D sites in the earlier studies, as nucleotides in coding regions may not be an ideal dataset because of codon usage bias and potential weak selection [34]. Regions that harbored large, low copy repeat sequences were excluded in this study to unambiguously determine the orthologous relationship. Such segmental duplicated regions may significantly inflate estimates of divergence due to non-orthologous sequence relationships [46,60] or gene conversion [55].

The dataset presented here, though much large than those used previously [2,12], is still a small part (0.4%) of the cattle, dog and human genomes. It is also worth noting that a number of the common assumptions made about neutral mutation, genetic drift, generation-time and population size, can affect these estimates [34,61], and rate calculations could be confounded by incorrect estimates of species divergence times. More comprehensive genome sequences and polymorphism data will be required to further clarify the important role of mutation rates in mammalian evolution. Further study of the molecular mechanisms behind mutation will be essential to understand the causes of mutation rate variation. Additional analyses will become feasible as the bovine genome approaches the finishing stage.

### Additional note

After the completion of this study, a comprehensive comparative analysis of the domestic dog genome reported similar genomic divergence estimates between dog and human [10].

## Conclusion

The unique features of this study include 1) optimal multiple (not pairwise) alignments were carefully constructed using a global (not local) alignment algorithm; 2) the scale was considerably larger as compared to earlier reports using small datasets of protein coding sequences or targeted genomic regions and 3) Our results were statistically significant and unbiased as supported by the mapping results of genome-wide randomly selected cattle BAC end sequences.

Therefore, this analysis provides a large-scale and unbiased assessment of genome divergences and regional variations of substitution rates among cattle, dog and human. Cattle had faster average rates of substitution as compared to dog and the difference was 6%. The global molecular clock needs to be adjusted to fit rates among mammalian species. These data will serve as a valuable baseline for future molecular evolution studies, especially in cattle and other livestock like sheep and pig.

## Methods

The comparative analyses performed in the current study were similar to those previously published [46]. However, several improvements to the previous analyses were 1) the use of three-way multiple sequence alignments instead of comparison of several pairwise alignments; 2) the application of REV rate matrices and ML methods using the PAML package [62] in addition to the simple K2 calculation; and 3) the optimization of alignment parameters and filter thresholds to deal with larger sequence divergences.

### Orthologous sequences

Large finished genomic sequences were retrieved from cattle BAC libraries (CH240 and RP42) from GenBank. Cattle sequence segments longer than 50 kb in length were then extracted and masked for common repeat elements [63,64]. Orthologous dog and human sequences were identified by sequence similarity searches [65] of cattle sequence queried against a formatted version of the assembled dog (canFam1, July 2004) and human (hg17, May 2004) genomes [32] using the following options (blastall -p blastn -U T -e 1e-05 -q -2 -r 1 -W 11 -G 3 -E 1 -b 25). Overlapping sequences within a species were excluded based on the genome assembly coordinates and sequence identity. We excluded any accession located within a known duplicated region of the human genome [60], because duplicated regions of the genome complicate identification of orthologous segments and confound genomic divergence estimates [18,46]. Because the assembly of the dog genome is based on only seven-fold "shotgun" sequence coverage, our analysis was limited to genomic sequences completely finished and containing no gaps or internal ambiguous bases. A total of 84 cattle clones and subclones (see Additional file 1 Table S3) met these criteria: 69 were generated by Baylor College of Medicine Human Genome Sequencing Center [66]; 12 were generated in National Institutes of Health Intramural Sequencing Center [67] as a part of a targeted comparative sequencing effort (the ENCODE – ENCyclopedia Of DNA Elements Project) [2,16,17]; and the remaining 3 were generated at the University of Oklahoma, Advanced Center for Genome Technology [68]. A complete list of all accessions, their consensus assemblies, their map locations with respect to the genomes of dog and human and their sequence attributes are provided (see Additional file 1 Table S3).

### Genomic sequence alignment

Orthologous sequences were extracted using parasight visualization software (J.A. Bailey, unpublished results) [69]. The mlagan algorithm [23] was used to construct all three-way multiple sequence global alignments. A subset of gap opening and gap extension penalties was chosen to minimize the frequency of both single-nucleotide substitution and insertion/deletion events in order to provide the most biologically meaningful optimal global alignment (See Results and Discussion). For equally parsimonious gap parameters, selected parameters (gap opening penalty of -1,000 and gap extension penalty of -10) were used so that known "young" transposition events were treated as a single insertion/deletion event. A total of 84 three-way multiple alignments for cattle, dog, and human (total alignment lengths ~15.5 Mb) were constructed with mlagan using ~10 Mb of genomic sequence from each species. All alignments were manually inspected for extreme fluctuations in genomic divergence. A suboptimal alignment was defined as any alignment which exceeded 3 standard deviations of the mean pairwise genomic divergence (window size 2 kb, slide 100 bp). These regions were considered separately in the analysis (Table 1). A total of 89 such subalignments were classified as suboptimal for cattle (732 kb), dog (619 kb), and human (822 kb). Only a small fraction (<5%) of all aligned bases was classified as suboptimal.

### Genomic divergence estimates

The branch lengths were calculated by maximum likelihood using version 3.15 of the PAML package, which allows base frequency change, all bases exchangeability and rate heterogeneity across sites (Table 1) [62]. The most general reversible substitution model (REV) was used (model = 7), rate variation among sites was allowed (fix_alpha = 0 and ncatG = 5), no molecular clock was assumed (clock = 0), unrooted trees were used, and ambiguity characters were discarded (cleandata = 1). Kimura's two-parameter (K2) method, which corrects for multiple events and transversion/transition mutational biases [70], was used to estimate genomic divergences in pairwise comparisons. Genomic divergences or branch lengths were always reported as the means ± their standard deviations. Insertion/deletion events were not factored into these calculations [71]. Coding, UTR, unique noncoding and repetitive regions from the sequence alignments were extracted using MaM (Multiple Alignment Manipulator) [36,72]. Repeat coordinates were identified using the slow option of RepeatMasker v3.0.5 with an updated RepBase library for cattle. Five major classes of repeats were considered in this analysis (LINEs, SINEs, LTR, DNA Transposons, and others). In order to eliminate the possibility that more divergent or novel common repeats may not have been effectively masked by RepeatMasker, intraspecific sequence-similarity searches were performed. Exon definition was limited to well-annoted human genes (NCBI RefSeq) [35,73]. Among these, a total of 1,909 exons corresponding to 193 genes were analyzed. The coding regions were extracted from exonic sequences between CDS start and end sites. The UTR regions contained both 5'-UTR (between transcription start and CDS start sites) and 3'- UTR (between transcription end and CDS end sites). Unique noncoding regions excluded both exonic and repetitive regions. Non-overlapping sliding window analyses (3-kp in Fig. 1, Additional file 1 FigS5A and 500-bp in Fig. S5B) were performed using align_slider (J.A. Bailey, unpublished results). Substitution rates were calculated from the LCA of cattle and dog using branch length/time assuming branch times of the cattle, dog and human lineages of 83, 83 and 101 mya, respectively [27,28]. All alignment attributes were maintained within a MySQL database which facilitated cross-referencing with various properties of the genomic sequence. Tajima's relative rate tests were performed on multiple alignments using MEGA3 [45]. ANOVA was performed to test variation in branch lengths of whole alignments or 3-kb nonoverlapping windows between and within autosomal chromosomes in cattle, dog, and human. Quadratic regression fits were implemented using the SigmaPlot software package.

## Abbreviations

AR: ancestral repeat

LCA: last common ancestor

mya: million years ago

Chr: Chromosome

BES: BAC end sequences

BTA: *Bos taurus *autosome

CFA: *Canis familiaris *autosome

HSA: *Homo sapiens *autosome

## Authors' contributions

GEL conceived and designed the experiments. GEL, CPVT, and LLS performed the experiments. GEL, CPVT, and TSS analyzed the data. GEL and LKM wrote the manuscript. All authors read and approved the final manuscript.

## Supplementary Material

Additional file 1 Adobe pdf format, this Supplemental Material file includes Figure S1. Optimization of Alignment Parameters. Figure S2. Cattle, Dog and Human Sequence and Alignment Properties. Table S3. Map Locations of Orthologous Trios in the Dog and Human Genome Assemblies. Table S4. Substitution Rates of Ancestral Repeats in Cattle, Dog and Human. Figure S5. Substitution Rate Variation. Click here for file

## Figures and Tables

**Table 1:** Nucleotide Divergence versus Sequence Class.

	**# loci**	**Total length (bp)**	**Aligned length (bp)**	**Tree length**	**Branch length**			Substitution rate* (change/site × 10^**-9**^)		
					
					**Cattle**	**Dog**	**Human**	**Cattle**	**Dog**	**Human**
**Overall**^†^	84	15507060	5521247	0.5265	0.1681 ± 0.0003	0.1547 ± 0.0003	0.2036 ± 0.0003	2.026 ± 0.003	1.864 ± 0.003	2.016 ± 0.003
**Overall-CG**	84	15507060	5282306	0.4984	0.1595 ± 0.0003	0.1451 ± 0.0002	0.1938 ± 0.0003	1.921 ± 0.003	1.748 ± 0.003	1.919 ± 0.003
**Coding**	52	137748	133235	0.1886	0.0644 ± 0.0010	0.0647 ± 0.0010	0.0595 ± 0.0009	0.776 ± 0.012	0.780 ± 0.012	0.589 ± 0.009
**UTR**	55	152130	115467	0.3855	0.1223 ± 0.0016	0.1472 ± 0.0018	0.1161 ± 0.0015	1.473 ± 0.019	1.773 ± 0.022	1.059 ± 0.010
**Unique noncoding**	84	9073616	4061797	0.5235	0.1676 ± 0.0003	0.1538 ± 0.0003	0.2021 ± 0.0004	2.019 ± 0.004	1.853 ± 0.003	2.001 ± 0.004
**Repetitive**	84	6409365	1157484	0.5719	0.1830 ± 0.0006	0.1668 ± 0.0006	0.2221 ± 0.0007	2.205 ± 0.007	2.010 ± 0.007	2.199 ± 0.007
**Repetitive-CG**	84	6409365	1112423	0.5460	0.1749 ± 0.0006	0.1581 ± 0.0006	0.2129 ± 0.0007	2.108 ± 0.007	1.905 ± 0.007	2.108 ± 0.007

Orthologous sequences were globally aligned with mlagan (Methods). A suboptimal alignment was defined as any alignment which exceeded 3 standard deviations of the mean K2 divergence (window size 2 kb, slide 100 bp). These regions were not included in the analysis. Coding sequence was restricted only to well-annotated human genes (NCBI RefSeq database). UTR regions included 5'- and 3'-UTRs. Repetitive sequences were detected using RepeatMasker (version 3.0.8). Unique noncoding (i.e. not annotated) regions excluded both exonic and repetitive regions. Due to the higher mutation rate of CpG dinucleotides, substitutions without CpG dinucleotides (Overall-CG, Repetitive-CG) were considered in each alignment.

* Substitution rate calculations assume branch times of the cattle, dog and human lineages from the LCA of cattle and dog of 83, 83 and 101 mya, respectively [27,28].

†: If suboptimal alignments were included in the analysis, the overall branch length increases to 0.1707 ± 0.0003, 0.1567 ± 0.0003 and 0.2069 ± 0.0004, respectively (Methods).
