# Supplementary material for: Genomic divergences among cattle, dog and human estimated from large-scale alignments of genomic sequences
Source: BMC Genomics. 2006 Jun 7;7:140. doi: 10.1186/1471-2164-7-140 (PMC1525190; doi:10.1186/1471-2164-7-140)
Supplement: Additional file 1 — Adobe pdf format, this Supplemental Material file includes Figure S1. Optimization of Alignment Parameters. Figure S2. Cattle, Dog and Human Sequence and Alignment Properties. Table S3. Map Locations of Orthologous Trios in the Dog and Human Genome Assemblies. Table S4. Substitution Rates of Ancestral Repeats in Cattle, Dog and Human. Figure S5. Substitution Rate Variation. [file 1471-2164-7-140-S1.pdf]

Supplemental Material

Figure S1. Optimization of Alignment Parameters.
Using 5 randomly chosen cattle-dog-human orthologous pairs (03: contig 03.03, 06: contig 05.06, 12: contig 09.12, 45: contig 38.45, 81: contig 57.81), we tested a range of gap-opening penalty (GOP: -1500, -1250, -1000, -750, -400, -200, -100) and gap-extension penalty parameters (GEP: -50, -25, -10, -5, -1). For each parameter combination, we considered the effect on sequence similarity, sequence similarity counting each indel as one mismatch, the number of mismatches, and the number of indels. We also considered the correspondence of gaps and the position of young retrotransposition events. Parameters were favored (gap-opening penalty of –1000 and gap-extension penalty of –10) which simultaneously reduced the number of indels and mismatches, and which treated new retrotransposition events as a single event.

Figure S2. Cattle, Dog and Human Sequence and Alignment Properties.
We obtained 84 qualified cattle-dog-human orthologous trios. For each alignment, a number of mutational properties such as branch length, pairwise REV ML distance, Kimura K2 distance, large indel frequency (>100 bp insertion/deletion event count per 10 kb) were compared. Other sequence properties such as GC%, SINE%, LINE% were also compared. Results varied within the expected levels of mammalian genome variation.

Table S3. Map Locations of Orthologous Trios in the Dog and Human Genome Assemblies.
A. Within 84 cattle genomic clones obtained, 20 of the sequences mapped to human phylogenetic group chromosome 7. B. Index of how the 16 merged fasta files were compiled is shown in the agp file format.

Table S4. Substitution Rates of Ancestral Repeats in Cattle, Dog and Human.
For each individual clone or contig, substitution rates were estimated by PAML from aligned ancestral repeats in cattle, dog and human. The average substitution rates were calculated for each chromosome according to the human genome placement.

Figure S5. Substitution Rate Variation.
Scatter plots of overall substitution rates determined from non-overlapping 3000-bp (A) and 500-bp (B) sliding windows for cattle-dog-human multiple sequence alignments. Suboptimal alignments were excluded. The means and their standard deviations are shown.

Figure S1. Optimization of Alignment Parameters.
Cattle-dog sequence alignments.

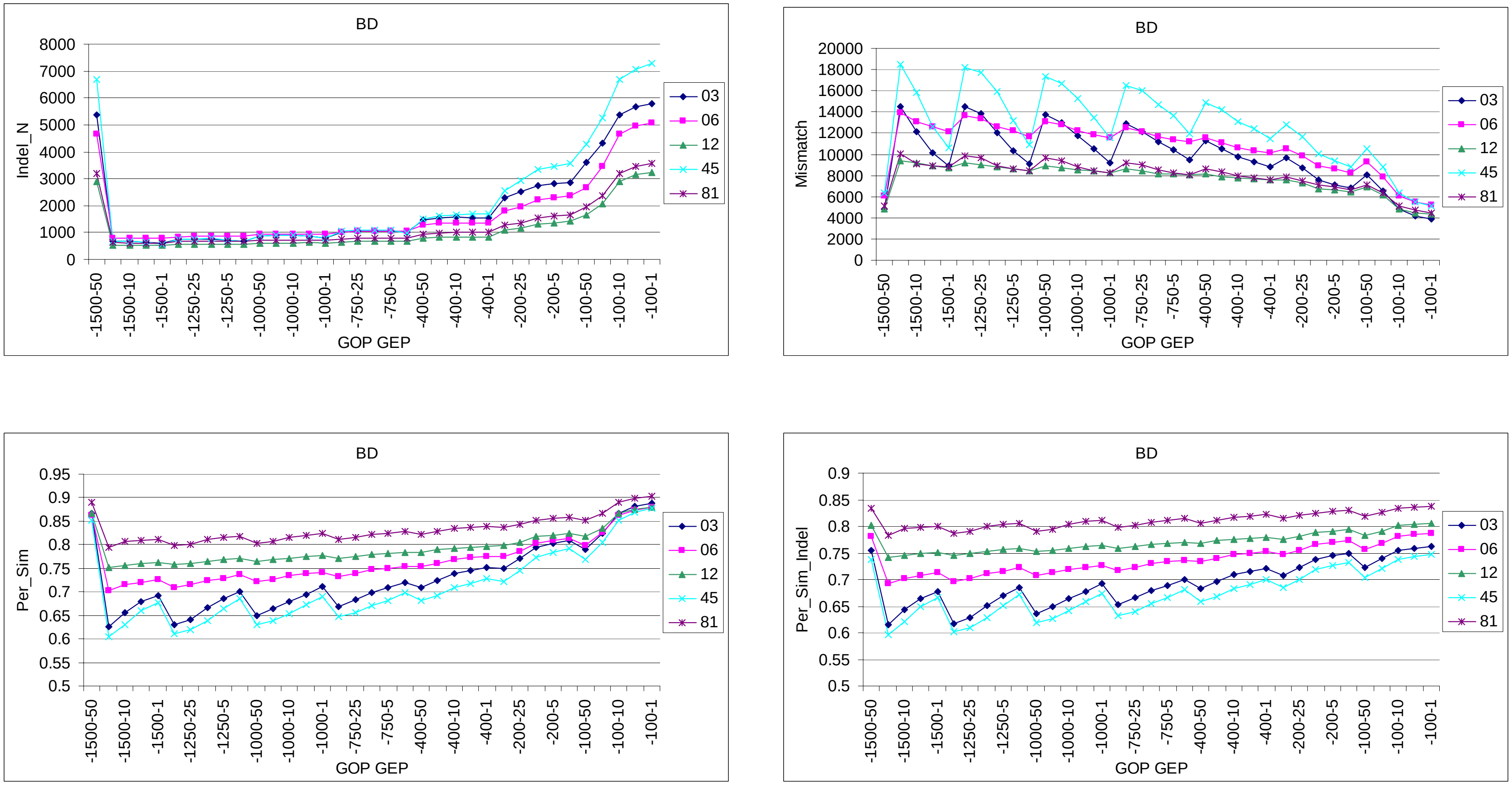

Cattle-human sequence alignments.

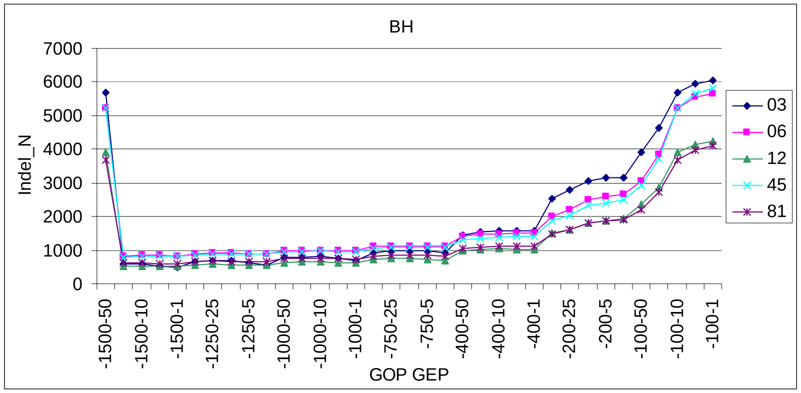

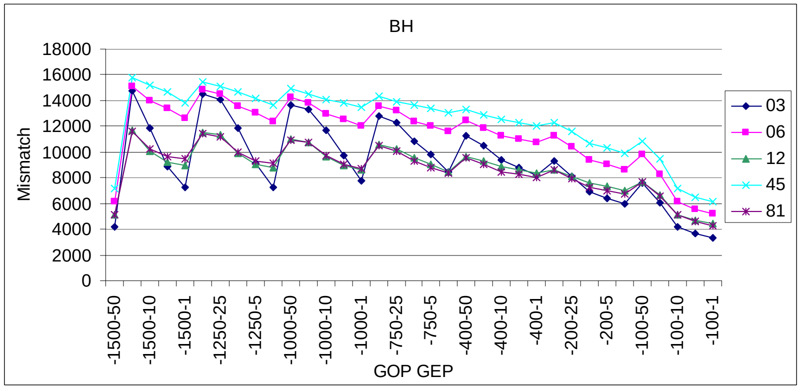

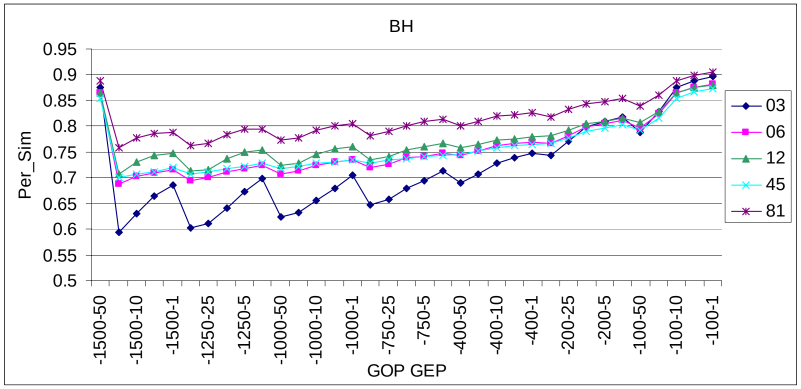

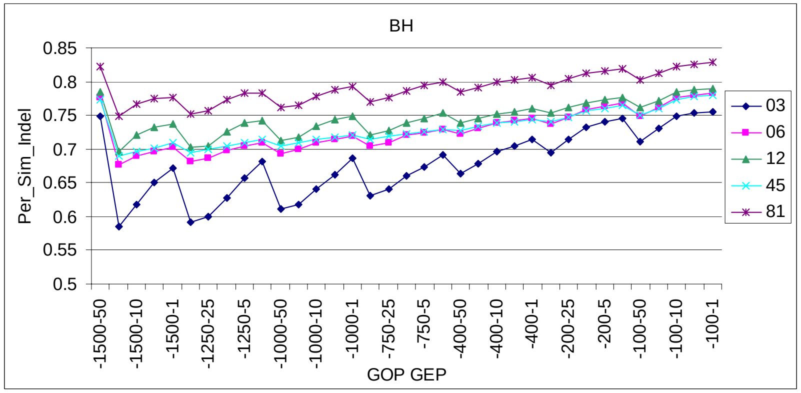

Dog-human sequence alignments.

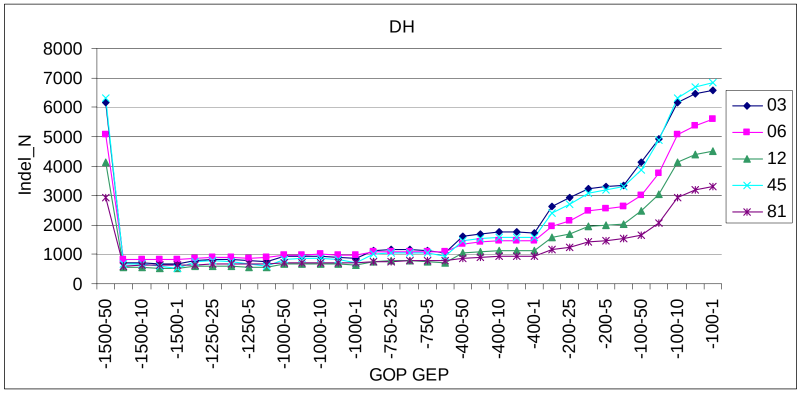

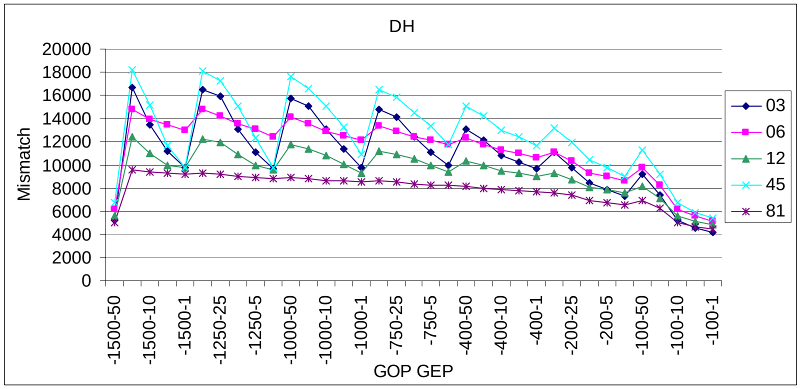

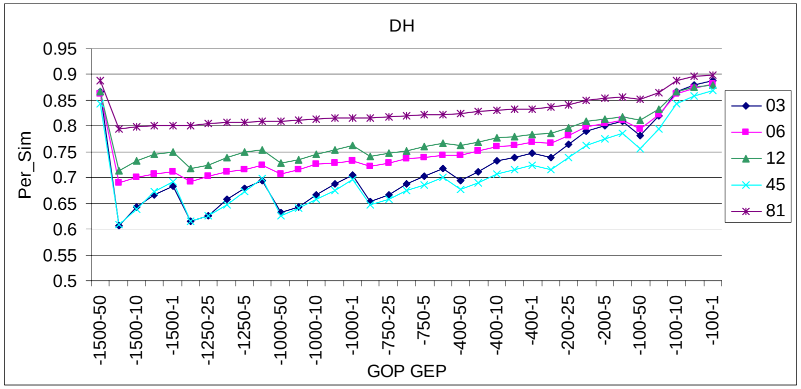

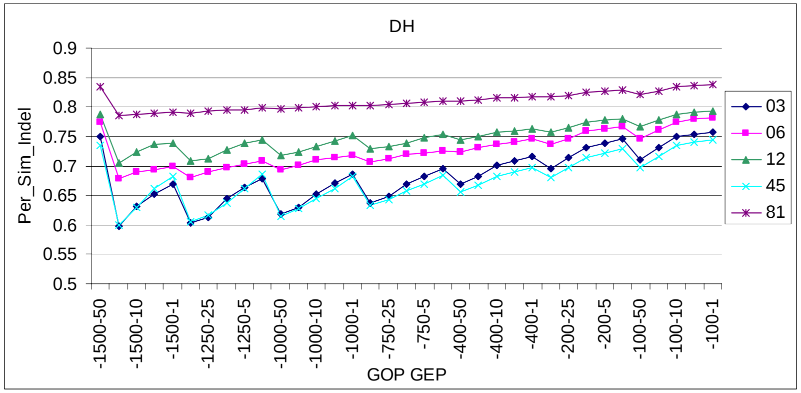

Figure S2. Cattle, Dog and Human Sequence and Alignment Properties.

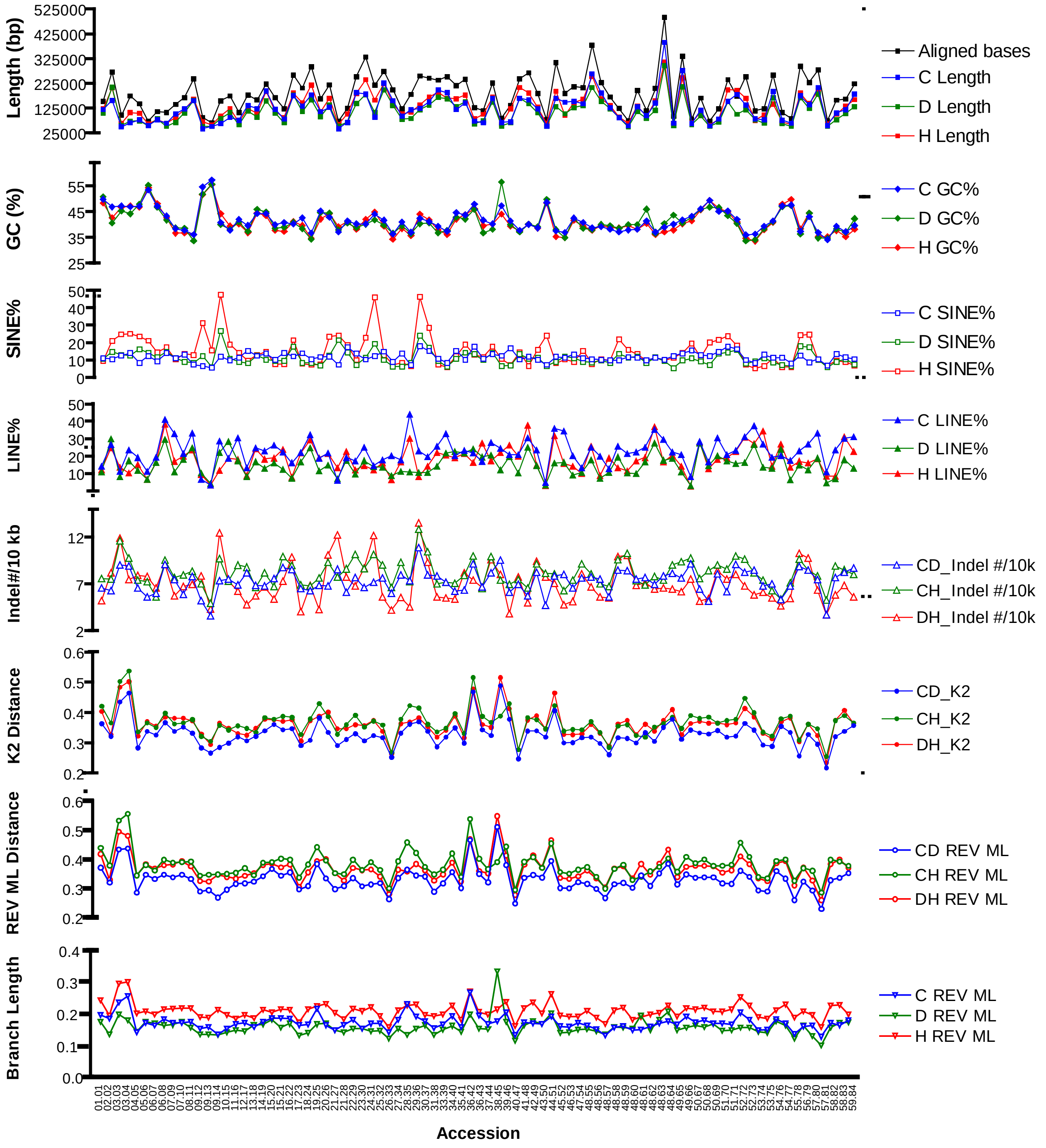

Table S3. Map Locations of Orthologous Trios in the Dog and Human Genome Assemblies.

A. Map Locations of Orthologous Trios

No.	Clones	Contig	Cattle Accession		Dog canFam1		Human hg17	
			Positions and Orientation		Positions and Orientation		Positions and Orientation	
1	RP42-459A17	01.01	AC092193.4:100919-221188	+	chr14:46029765-46135257	+	chr7:30585342-30707957	+
2	rp42-51p13	02.02	AC103584.15:4869-162078	-	chr26:26334081-26543457	+	chr22:28760476-28966885	+
3	rp42-513g13	03.03	AC107065.5:32394-83065	+	chr25:29108406-29051991	-	chr8:11698086-11762835	+
4	rp42-513g13	03.04	AC107065.5:83274-150215	+	chr25:29051337-28977709	-	chr8:11763095-11870558	+
5	RP42-518P7	04.05	AC129959.6:131594-209300	+	chr9:17538931-17613044	+	chr17:44046521-44151615	+
6	RP42-221D7	05.06	AC136966.2:61682-116581	-	chr9:18604343-18658651	+	chr17:45339353-45401801	+
7	CH240-60O13	06.07	AC149665.2:71434-149517	-	chr11:22065731-22148254	+	chr5:131774510-131854613	+
8	CH240-60O13	06.08	AC149665.2:1796-66637	-	chr11:22153608-22206679	+	chr5:131859832-131923598	+
9	CH240-57J21	07.09	AC149669.2:124272-226591	-	chr5:89383193-89314559	-	chr16:60818827-60906505	+
10	CH240-57J21	07.10	AC149669.2:333-122665	-	chr5:89311839-89205021	-	chr16:60906795-61025689	+
11	CH240-248M14	08.11	AC149720.2:11424-167172	+	chr25:9747993-10391281	+	chr13:32082240-32244382	+
12	CH240-361I2	09.12	AC149756.4:130172-173471	-	chr20:42342815-42290653	-	chr3:49792479-49862644	+
13	CH240-361I2	09.13	AC149756.4:73985-125954	-	chr20:42285949-42233885	-	chr3:49868199-49925447	+
14	CH240-361I2	09.14	AC149756.4:6067-68813	-	chr20:42218256-42135476	-	chr3:49940788-50034633	+
15	CH240-53K11	10.15	AC149763.5:60855-148984	+	chr25:10785849-10678516	-	chr13:31775704-31899466	+
16	CH240-12E22	11.16	AC149769.2:3063-80460	+	chr13:20515866-20574455	+	chr8:119213679-119283057	+
17	CH240-9E7	12.17	AC149778.2:748-136557	+	chr7:62809814-63496620	+	chr18:23782859-23905738	+
18	CH240-5F6	13.18	AC149779.3:1294-125301	-	chr24:22701407-22611789	-	chr20:1137105-1242156	+
19	CH240-6M21	14.19	AC149782.2:638-196267	+	chr34:22075090-22229562	+	chr3:187540018-187733482	+
20	CH240-3K17	15.20	AC149783.2:32-121081	+	chr7:63844657-63738049	-	chr18:23519681-23632424	+
21	CH240-3K17	15.21	AC149783.2:121141-202657	+	chr7:63735650-63668564	-	chr18:23632488-23720071	+
22	CH240-308E11	16.22	AC150482.2:2477-177631	-	chr20:28377373-28198847	-	chr3:65775307-65961148	+
23	CH240-466B8	17.23	AC150488.2:985-134509	-	chr14:19660232-19772043	+	chr7:90595511-90741190	+
24	CH240-454H24	18.24	AC150492.2:2310-181487	+	chr14:27800487-27959739	+	chr7:9840649-10059060	+
25	CH240-105M18	19.25	AC150499.3:92163-203536	-	chr5:6769079-6677660	-	chr11:130586482-130697937	+
26	CH240-319N17	20.26	AC150512.3:1350-129979	+	chr3:65262399-65925035	+	chr4:17282199-17447839	+
27	CH240-161G19	21.27	AC150517.3:37210-80100	+	chr13:51786601-51840848	+	chr4:57152301-57212925	+
28	CH240-161G19	21.28	AC150517.3:111912-180359	+	chr13:51884839-51953168	+	chr4:57254710-57356795	+
29	CH240-89P8	22.29	AC150524.3:362-188772	+	chr1:22891628-23548122	+	chr18:50581215-50766301	+
30	CH240-86O22	23.30	AC150530.4:1821-183948	+	chr12:67878733-68058426	+	chr6:108233325-108473815	+

31	CH240-382E8	24.31	AC150542.2:1139-89971	+	chr1:32238245-32479376	+	chr22:30070900-30228829	+
32	CH240-184G17	25.32	AC150561.4:12-227300	+	chr13:20618770-20818692	+	chr8:119333111-119558332	+
33	CH240-223K16	26.33	AC150569.2:6411-162071	+	chr14:56306856-56442732	+	chr7:113799894-113940178	+
34	CH240-238N22	27.34	AC150573.4:71-92671	-	chr1:15978104-15897283	-	chr18:59979761-60081474	+
35	CH240-237H4	28.35	AC150574.2:16524-135281	+	chr7:50215621-50132127	-	chr18:39047293-39157065	+
36	CH240-268P24	29.36	AC150577.4:605-125168	-	chr12:38447781-38566611	+	chr6:74201469-74340188	+
37	CH240-448P19	30.37	AC150635.4:651-151762	-	chr20:25390466-25252853	-	chr3:69344360-69513654	+
38	CH240-223I2	31.38	AC150644.4:1043-199457	-	chr14:11872632-11704042	-	chr7:126130176-126318814	+
39	CH240-443I13	33.39	AC150674.4:3633-192027	-	chr14:56758901-56922307	+	chr7:114308522-114473710	+
40	CH240-451M14	34.40	AC150675.2:51176-171337	-	chr17:14911000-15043125	+	chr2:16338506-16502117	+
41	CH240-151A24	35.41	AC150690.7:606-147383	-	chr21:34626826-34777799	+	chr11:8992079-9170005	+
42	CH240-275I24	36.42	AC150707.3:39753-112994	-	chr25:12866898-12804534	-	chr13:29591596-29674106	+
43	CH240-275I24	36.43	AC150707.3:791-67489	-	chr25:12399029-12731304	+	chr13:29650317-29750771	+
44	CH240-288E20	37.44	AC150752.4:682-165588	-	chr13:51490153-51641017	+	chr4:56813280-56981839	+
45	CH240-283G19	38.45	AC150753.3:51450-119516	-	chr15:11052670-10999201	-	chr1:33820824-33883038	+
46	CH240-472P12	39.46	AC150855.6:36600-107393	+	chr1:16184688-16117617	-	chr18:59686006-59808307	+
47	CH240-431L22	40.47	AC150858.4:407-165819	+	chr14:42581267-42748157	+	chr7:26592673-26801337	+
48	CH240-316E7	41.48	AC150871.4:6457-164609	-	chr19:34524774-35180988	+	chr2:117975790-118162677	+
49	CH240-491H11	42.49	AC150879.3:379-122583	-	chr31:36217864-36325326	+	chr21:39445543-39574172	+
50	CH240-483B20	43.50	AC150887.4:57772-111059	-	chr37:27886769-27939902	+	chr2:218907788-218972107	+
51	CH240-475A10	44.51	AC150888.5:3021-168040	+	chr26:36002667-36670894	+	chr10:54950334-55144694	+
52	CH240-186K21	45.52	AC150993.4:32482-181986	-	chr27:16358967-16256152	-	chr12:38934819-39032016	+
53	CH240-293H4	46.53	AC151060.4:437-153612	-	chr37:29316225-29444020	+	chr2:220577954-220725201	+
54	CH240-106N15	47.54	AC151132.3:27576-170686	+	chr31:30308504-30445036	+	chr21:32893422-33055419	+
55	RP42	48.55	cow_01:85480-348895	+	chr14:57799075-58007894	+	chr7:115480168-115733738	+
56	RP42	48.56	cow_01:355838-542123	+	chr14:58015451-58168204	+	chr7:115742150-115905575	+
57	RP42	48.57	cow_01:543889-670944	+	chr14:58168824-58291520	+	chr7:115907285-116042703	+
58	RP42	48.58	cow_01:733214-820313	+	chr14:58333594-58421805	+	chr7:116097634-116187129	+
59	RP42	48.59	cow_01:822336-876202	+	chr14:58423892-58474186	+	chr7:116189100-116253984	+
60	RP42	48.60	cow_01:876855-1007769	+	chr14:58474644-58586519	+	chr7:116254629-116389704	+
61	RP42	48.61	cow_01:1009037-1104164	+	chr14:58591386-58675495	+	chr7:116391065-116482842	+
62	RP42	48.62	cow_01:1172107-1319065	+	chr14:58742655-58859228	+	chr7:116558438-116713593	+
63	RP42	48.63	cow_01:1319161-1710055	+	chr14:58859726-59155670	+	chr7:116713683-117025856	+
64	RP42	48.64	cow_01:1710263-1774889	+	chr14:59156241-59211767	+	chr7:117026088-117090776	+
65	CH240	49.65	cow_05:1211-278261	+	chr13:20088716-20300722	+	chr8:118731516-118978613	+
66	CH240	49.66	cow_05:278374-341917	+	chr13:20301496-20360734	+	chr8:118979511-119046512	+
67	CH240	50.67	cow_06:2735-119317	+	chr5:19943353-19848448	-	chr11:115953186-116065379	+

68	CH240	50.68	cow_06:124178-178472	+	chr5:19842956-19790929	-	chr11:116072131-116127882	+
69	CH240	50.69	cow_06:178942-260254	+	chr5:19788444-19719670	-	chr11:116128658-116209756	+
70	CH240	51.70	cow_08:94-153739	+	chr10:33870374-33718167	-	chr22:31579088-31778013	+
71	CH240	51.71	cow_08:154879-336475	+	chr10:33717034-33616479	-	chr22:31779065-31976875	+
72	CH240	52.72	cow_09:28256-165788	+	chr10:56341700-56460458	+	chr2:51642204-51807175	+
73	CH240	52.73	cow_09:175901-259328	+	chr10:56465778-56544300	+	chr2:51808934-51886339	+
74	CH240	53.74	cow_10:797-78322	+	chr8:32759064-32824221	+	chr14:53308621-53406357	+
75	CH240	53.75	cow_10:80935-273626	+	chr8:32825669-32993955	+	chr14:53408508-53550801	+
76	CH240	54.76	cow_11:191125-268498	-	chr11:21628965-21691998	+	chr5:131350907-131418895	+
77	CH240	54.77	cow_11:120648-185445	-	chr11:21693757-21746116	+	chr5:131420683-131479783	+
78	CH240	55.78	cow_12:24634-200470	+	chrX:98470472-98638698	+	chrX:122800129-122986208	+
79	CH240	56.79	cow_13:126517-265691	+	chr4:25935619-26060162	+	chr10:73478384-73621937	+
80	CH240	57.80	cow_14:63260-272010	-	chr14:55957811-56140762	+	chr7:113414403-113609271	+
81	CH240	57.81	cow_14:2551-58567	-	chr14:56143921-56196551	+	chr7:113612734-113675142	+
82	CH240	58.82	cow_15:4368-110889	+	chrX:79450003-79882515	+	chr16:61254379-61354977	+
83	CH240	58.83	cow_15:111211-230644	+	chr5:88971363-88867442	-	chr16:61355350-61489892	+
84	RP42	59.84	cow_16:2220-185260	+	chr14:59238068-59368896	+	chr7:117118972-117278620	+

B. Index of compilation of 16 assembled fasta files.

Contigs	Begin	End	Part No	Type	Accession	Acc Begin	Acc End	Orientation	Clones
cow_01	1	120491	1	F	AC146966.3	1	120491	+	RP42-139G14
cow_01	120492	222858	2	F	AC156365.3	1	102367	+	RP42-18L9
cow_01	222859	382810	3	F	AC090976.2	1	159952	+	RP42-400M23
cow_01	382811	462244	4	F	AC090961.2	15011	94444	+	RP42-552E5
cow_01	462245	600007	5	F	AC087843.2	19403	157165	+	RP42-550C12
cow_01	600008	794202	6	F	AC087860.2	9502	203696	+	RP42-135P2
cow_01	794203	915759	7	F	AC089991.2	37640	159196	+	RP42-194K3
cow_01	915760	1081208	8	F	AC090031.2	48393	213841	+	RP42-341K3
cow_01	1081209	1097160	9	F	AC122112.2	3689	19640	+	RP42-332L24
cow_01	1097161	1259903	10	F	AC091728.2	1	162743	+	RP42-99M11
cow_01	1259904	1376087	11	F	AC089992.2	31739	147922	+	RP42-210F11
cow_01	1376088	1529357	12	F	AC089993.2	9590	162859	+	RP42-553M7
cow_01	1529358	1703603	13	F	AC091252.3	9138	183383	+	RP42-324C24
cow_01	1703604	1775989	14	F	AC156366.3	19336	91721	+	RP42-304J18
cow_02	1	216538	1	F	AC092496.3	1	216538	+	RP42-394P20
cow_02	216539	355780	2	F	AC091660.2	33827	173068	+	RP42-354B6
cow_03	1	43157	1	F	AC098686.11	164686	121530	-	rp42-146c17
cow_03	43158	234878	2	F	AC096629.10	4	191724	+	rp42-152a4
cow_03	234879	365613	3	F	AC092858.21	34856	165590	+	rp42-147e22
cow_04	1	199710	1	F	AC151117.4	1	199710	+	CH240-85N4
cow_04	199711	274841	2	F	AC149645.4	75131	1	-	CH240-50L4
cow_05	1	116055	1	F	AC150847.5	134331	18277	-	CH240-372I17
cow_05	116056	291051	2	F	AC150881.4	1	174996	+	CH240-354J15
cow_05	291052	372536	3	F	AC149676.2	86932	168416	+	CH240-43C16
cow_06	1	65576	1	F	AC150916.3	158580	93005	-	CH240-309L24
cow_06	65577	261763	2	F	AC149679.2	1	196187	+	CH240-27A8
cow_07	1	176832	1	F	AC149683.2	1	176832	+	CH240-493H15
cow_07	176833	281368	2	F	AC149717.2	104536	1	-	CH240-194N8
cow_08	1	151547	1	F	AC149762.2	187366	35820	-	CH240-123C23
cow_08	151548	337665	2	F	AC149694.2	1	186118	+	CH240-423C21
cow_09	1	171707	1	F	AC149716.2	1	171707	+	CH240-31B10
cow_09	171708	281423	2	F	AC150532.2	109716	1	-	CH240-105A12
cow_10	1	126878	1	F	AC150687.3	144223	17346	-	CH240-243M2
cow_10	126879	313794	2	F	AC149774.2	1	186916	+	CH240-10G15
cow_11	1	171213	1	F	AC150515.3	1	171213	+	CH240-118E9
cow_11	171214	268771	2	F	AC150860.4	97558	1	-	CH240-424I20
cow_12	1	88090	1	F	AC150653.5	158246	70157	-	CH240-226L9
cow_12	88091	252010	2	F	AC150694.2	1	163920	+	CH240-144A17
cow_13	1	223559	1	F	AC150516.2	237116	13558	-	CH240-117L9
cow_13	223560	394470	2	F	AC150866.2	1	170911	+	CH240-290G4
cow_14	1	203152	1	F	AC150546.2	1	203152	+	CH240-77D21
cow_14	203153	273777	2	F	AC150593.2	70625	1	-	CH240-105D16
cow_15	1	158286	1	F	AC150664.2	1	158286	+	CH240-244O10
cow_15	158287	232609	2	F	AC150919.6	74323	1	-	CH240-303F16
cow_16	1	78774	1	F	AC156367.3	1	78774	+	RP42-394P24
cow_16	78775	246694	2	F	AC146967.3	18692	186611	+	RP42-279M12

Table S4. *Substitution Rates of Ancestral Repeats in Cattle, Dog and Human.*

Contig	Human hg17 Positions and Orientation	Alignment Length	Substitution Rate (X10^{-9}change/site/year) Cattle	Dog	Human
38.45	chr1:33820824-33883038	86127	2.120 ± 0.082	3.860 ± 0.133	2.191 ± 0.080
	**Subtotal**	**86127**	**2.120 ± 0.082**	**3.860 ± 0.133**	**2.191 ± 0.080**
34.40	chr2:16338506-16502117	216364	2.428 ± 0.061	2.087 ± 0.054	2.367 ± 0.058
52.72	chr2:51642204-51807175	251466	2.357 ± 0.060	2.041 ± 0.053	2.213 ± 0.054
52.73	chr2:51808934-51886339	114557	2.140 ± 0.135	2.320 ± 0.142	2.301 ± 0.140
41.48	chr2:117975790-118162677	267904	2.051 ± 0.046	1.853 ± 0.042	2.014 ± 0.044
43.50	chr2:218907788-218972107	78663	2.606 ± 0.077	2.094 ± 0.066	2.724 ± 0.078
46.53	chr2:220577954-220725201	212712	2.714 ± 0.123	2.312 ± 0.110	2.661 ± 0.118
	**Subtotal**	**1141666**	**2.340 ± 0.028**	**2.036 ± 0.025**	**2.308 ± 0.027**
09.12	chr3:49792479-49862644	86717	2.075 ± 0.049	1.998 ± 0.048	2.081 ± 0.049
09.13	chr3:49868199-49925447	66960	2.520 ± 0.184	1.611 ± 0.136	2.802 ± 0.198
09.14	chr3:49940788-50034633	154663	1.946 ± 0.133	1.931 ± 0.128	2.274 ± 0.145
16.22	chr3:65775307-65961148	258203	1.751 ± 0.070	1.766 ± 0.070	2.910 ± 0.069
30.37	chr3:69344360-69513654	246792	2.382 ± 0.061	2.348 ± 0.060	2.350 ± 0.058
14.19	chr3:187540018-187733482	223181	2.255 ± 0.060	2.131 ± 0.058	2.187 ± 0.057
	**Subtotal**	**1036516**	**2.164 ± 0.029**	**2.078 ± 0.028**	**2.291 ± 0.029**
20.26	chr4:17282199-17447839	219368	2.153 ± 0.060	1.981 ± 0.057	2.378 ± 0.063
37.44	chr4:56813280-56981839	227041	2.086 ± 0.059	2.069 ± 0.058	2.111 ± 0.057
21.27	chr4:57152301-57212925	73590	2.051 ± 0.131	2.090 ± 0.134	2.031 ± 0.127
21.28	chr4:57254710-57356795	124192	2.060 ± 0.078	1.852 ± 0.072	1.862 ± 0.069
	**Subtotal**	**644191**	**2.105 ± 0.036**	**1.993 ± 0.035**	**2.150 ± 0.035**
54.76	chr5:131350907-131418895	107648	2.202 ± 0.087	2.177 ± 0.086	2.121 ± 0.081
54.77	chr5:131420683-131479783	82828	1.830 ± 0.087	2.180 ± 0.099	2.261 ± 0.099
06.07	chr5:131774510-131854613	111195	1.998 ± 0.061	2.075 ± 0.063	1.975 ± 0.058
06.08	chr5:131859832-131923598	108131	2.294 ± 0.080	1.996 ± 0.071	2.279 ± 0.075
	**Subtotal**	**409802**	**2.089 ± 0.039**	**2.089 ± 0.037**	**2.126 ± 0.037**
29.36	chr6:74201469-74340188	255568	2.064 ± 0.057	1.893 ± 0.053	2.366 ± 0.061
23.30	chr6:108233325-108473815	331897	2.577 ± 0.096	2.200 ± 0.087	2.698 ± 0.097
	**Subtotal**	**587465**	**2.219 ± 0.049**	**1.986 ± 0.046**	**2.467 ± 0.052**
18.24	chr7:9840649-10059060	292357	1.969 ± 0.054	1.834 ± 0.051	2.150 ± 0.055
40.47	chr7:26592673-26801337	243369	2.408 ± 0.127	1.583 ± 0.094	1.994 ± 0.105
01.01	chr7:30585342-30707957	153592	2.371 ± 0.080	1.788 ± 0.065	2.020 ± 0.068
17.23	chr7:90595511-90741190	206463	2.237 ± 0.060	1.942 ± 0.054	2.232 ± 0.057
57.80	chr7:113414403-113609271	278912	2.265 ± 0.058	2.007 ± 0.052	2.223 ± 0.054
57.81	chr7:113612734-113675142	75813	1.986 ± 0.049	1.840 ± 0.047	1.955 ± 0.047
26.33	chr7:113799894-113940178	199486	1.851 ± 0.058	1.864 ± 0.057	1.915 ± 0.056
33.39	chr7:114308522-114473710	251364	1.998 ± 0.081	2.092 ± 0.083	2.264 ± 0.086
48.55	chr7:115480168-115733738	379737	2.216 ± 0.124	1.788 ± 0.105	2.656 ± 0.144
48.56	chr7:115742150-115905575	228138	1.933 ± 0.060	1.929 ± 0.060	1.812 ± 0.055
48.57	chr7:115907285-116042703	170372	2.023 ± 0.072	2.584 ± 0.086	2.151 ± 0.073
48.58	chr7:116097634-116187129	124172	1.983 ± 0.053	1.916 ± 0.051	2.002 ± 0.050
48.59	chr7:116189100-116253984	74878	2.260 ± 0.047	2.282 ± 0.047	2.248 ± 0.046
48.60	chr7:116254629-116389704	197714	2.351 ± 0.084	2.457 ± 0.086	2.303 ± 0.080
48.61	chr7:116391065-116482842	114800	2.461 ± 0.072	2.227 ± 0.065	2.085 ± 0.060
48.62	chr7:116558438-116713593	205213	2.047 ± 0.051	1.735 ± 0.046	2.181 ± 0.051
48.63	chr7:116713683-117025856	491059	1.867 ± 0.055	1.558 ± 0.049	1.856 ± 0.052
48.64	chr7:117026088-117090776	93027	2.386 ± 0.066	2.230 ± 0.064	2.367 ± 0.064
59.84	chr7:117118972-117278620	223355	2.258 ± 0.070	1.745 ± 0.059	1.945 ± 0.061
31.38	chr7:126130176-126318814	238636	2.236 ± 0.071	1.860 ± 0.063	2.388 ± 0.072

	**Subtotal**	**4242457**	**2.142 ± 0.014**	**1.976 ± 0.013**	**2.126 ± 0.014**
03.03	chr8:11698086-11762835	97323	3.300 ± 0.173	2.371 ± 0.133	3.165 ± 0.165
03.04	chr8:11763095-11870558	174511	3.104 ± 0.147	2.470 ± 0.123	2.848 ± 0.134
49.65	chr8:118731516-118978613	333441	2.098 ± 0.042	1.863 ± 0.039	2.021 ± 0.041
49.66	chr8:118979511-119046512	81166	2.486 ± 0.108	1.936 ± 0.098	2.280 ± 0.094
11.16	chr8:119213679-119283057	108845	2.092 ± 0.067	1.992 ± 0.065	2.001 ± 0.059
25.32	chr8:119333111-119558332	273031	2.151 ± 0.045	1.858 ± 0.040	2.099 ± 0.043
	**Subtotal**	**1068317**	**2.258 ± 0.028**	**1.948 ± 0.024**	**2.169 ± 0.026**
44.51	chr10:54950334-55144694	308959	2.655 ± 0.102	2.610 ± 0.099	2.987 ± 0.112
56.79	chr10:73478384-73621937	228934	2.202 ± 0.067	2.286 ± 0.070	2.350 ± 0.069
	**Subtotal**	**537893**	**2.372 ± 0.057**	**2.417 ± 0.058**	**2.583 ± 0.059**
35.41	chr11:8992079-9170005	242038	2.100 ± 0.052	1.969 ± 0.048	2.011 ± 0.048
50.67	chr11:115953186-116065379	166340	2.163 ± 0.095	1.618 ± 0.077	2.012 ± 0.085
50.68	chr11:116072131-116127882	73399	2.070 ± 0.067	1.972 ± 0.065	2.040 ± 0.064
50.69	chr11:116128658-116209756	123764	2.669 ± 0.084	2.084 ± 0.070	2.342 ± 0.074
19.25	chr11:130586482-130697937	163201	1.983 ± 0.045	1.836 ± 0.042	1.877 ± 0.042
	**Subtotal**	**768742**	**2.151 ± 0.028**	**1.916 ± 0.025**	**2.022 ± 0.025**
45.52	chr12:38934819-39032016	185076	2.207 ± 0.063	1.776 ± 0.054	2.080 ± 0.056
	**Subtotal**	**185076**	**2.207 ± 0.073**	**1.776 ± 0.060**	**2.080 ± 0.069**
36.42	chr13:29591596-29674106	127145	2.955 ± 0.114	2.201 ± 0.090	2.611 ± 0.100
36.43	chr13:29650317-29750771	115102	2.705 ± 0.111	2.093 ± 0.090	2.283 ± 0.095
10.15	chr13:31775704-31899466	174749	2.128 ± 0.075	1.899 ± 0.069	2.158 ± 0.072
08.11	chr13:32082240-32244382	244312	2.304 ± 0.064	2.019 ± 0.059	2.347 ± 0.063
	**Subtotal**	**661308**	**2.451 ± 0.042**	**2.039 ± 0.037**	**2.337 ± 0.040**
53.74	chr14:53308621-53406357	123858	1.977 ± 0.064	1.901 ± 0.063	2.141 ± 0.059
53.75	chr14:53408508-53550801	258669	2.053 ± 0.055	1.890 ± 0.052	2.015 ± 0.052
	**Subtotal**	**382527**	**2.018 ± 0.043**	**1.888 ± 0.041**	**2.055 ± 0.043**
07.09	chr16:60818827-60906505	140525	2.286 ± 0.087	2.236 ± 0.084	2.224 ± 0.081
07.10	chr16:60906795-61025689	167605	2.337 ± 0.086	2.300 ± 0.082	2.261 ± 0.080
58.82	chr16:61254379-61354977	158049	2.495 ± 0.100	2.169 ± 0.088	2.689 ± 0.104
58.83	chr16:61355350-61489892	162397	2.217 ± 0.072	2.445 ± 0.076	2.599 ± 0.070
	**Subtotal**	**628576**	**2.323 ± 0.043**	**2.290 ± 0.042**	**2.433 ± 0.044**
04.05	chr17:44046521-44151615	142683	1.945 ± 0.086	1.863 ± 0.083	2.47 ± 0.100
05.06	chr17:45339353-45401801	73205	2.095 ± 0.086	2.061 ± 0.084	1.833 ± 0.073
	**Subtotal**	**215888**	**2.011 ± 0.059**	**1.947 ± 0.058**	**2.128 ± 0.059**
15.20	chr18:23519681-23632424	167556	2.380 ± 0.081	2.443 ± 0.084	2.139 ± 0.071
15.21	chr18:23632488-23720071	123625	2.659 ± 0.095	2.120 ± 0.080	2.224 ± 0.079
12.17	chr18:23782859-23905738	177527	2.376 ± 0.076	2.036 ± 0.069	2.156 ± 0.068
28.35	chr18:39047293-39157065	181140	3.227 ± 0.127	1.812 ± 0.100	2.621 ± 0.104
22.29	chr18:50581215-50766301	252513	2.481 ± 0.072	2.013 ± 0.061	2.445 ± 0.069
39.46	chr18:59686006-59808307	135578	2.871 ± 0.142	2.234 ± 0.114	2.688 ± 0.133
27.34	chr18:59979761-60081474	122499	2.510 ± 0.113	2.336 ± 0.107	2.489 ± 0.111
	**Subtotal**	**1160438**	**2.546 ± 0.035**	**2.137 ± 0.031**	**2.331 ± 0.032**
13.18	chr20:1137105-1242156	158641	1.895 ± 0.054	1.922 ± 0.054	1.929 ± 0.052
	**Subtotal**	**158641**	**1.895 ± 0.054**	**1.922 ± 0.054**	**1.929 ± 0.052**
47.54	chr21:32893422-33055419	207697	2.199 ± 0.067	1.937 ± 0.061	2.202 ± 0.065
42.49	chr21:39445543-39574172	184694	2.286 ± 0.086	2.410 ± 0.089	2.835 ± 0.101
	**Subtotal**	**392391**	**2.241 ± 0.053**	**2.135 ± 0.052**	**2.462 ± 0.055**
02.02	chr22:28760476-28966885	271443	2.224 ± 0.048	1.758 ± 0.041	1.961 ± 0.043
24.31	chr22:30070900-30228829	217929	2.189 ± 0.087	1.873 ± 0.078	2.861 ± 0.106
51.70	chr22:31579088-31778013	240638	2.229 ± 0.042	1.764 ± 0.035	2.121 ± 0.039
51.71	chr22:31779065-31976875	174254	2.080 ± 0.053	1.799 ± 0.048	2.117 ± 0.051
	**Subtotal**	**904264**	**2.190 ± 0.025**	**1.781 ± 0.023**	**2.141 ± 0.025**

55.78	chrX:122800129-122986208	294775	1.771 ± 0.045	1.680 ± 0.043	2.083 ± 0.049
	**Subtotal**	**294775**	**1.771 ± 0.045**	**1.680 ± 0.043**	**2.083 ± 0.049**
	**Total**	**15507060**	**2.205 ± 0.007**	**2.010 ± 0.007**	**2.199 ± 0.007**

Figure S5. Substitution Rate Variation

A.

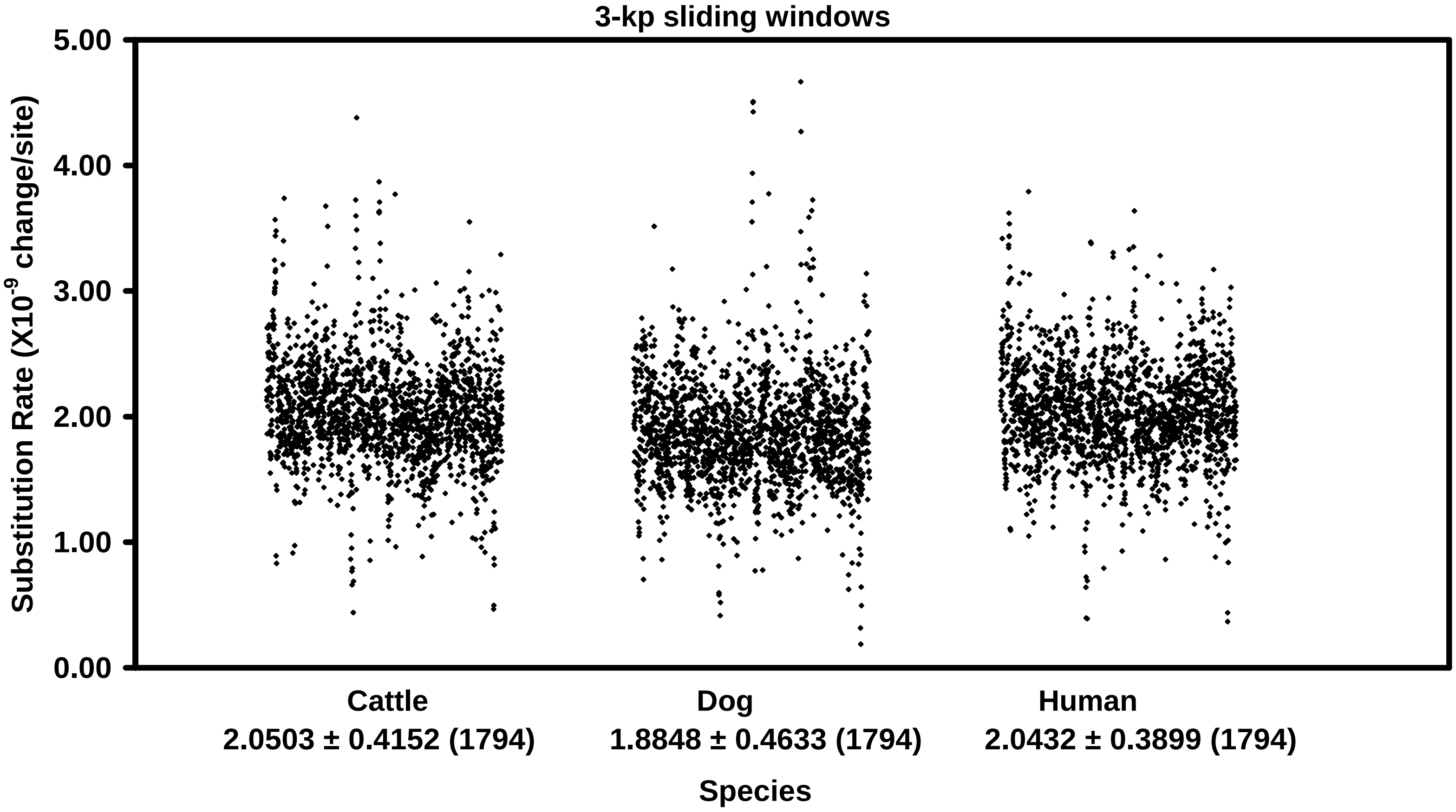

B.

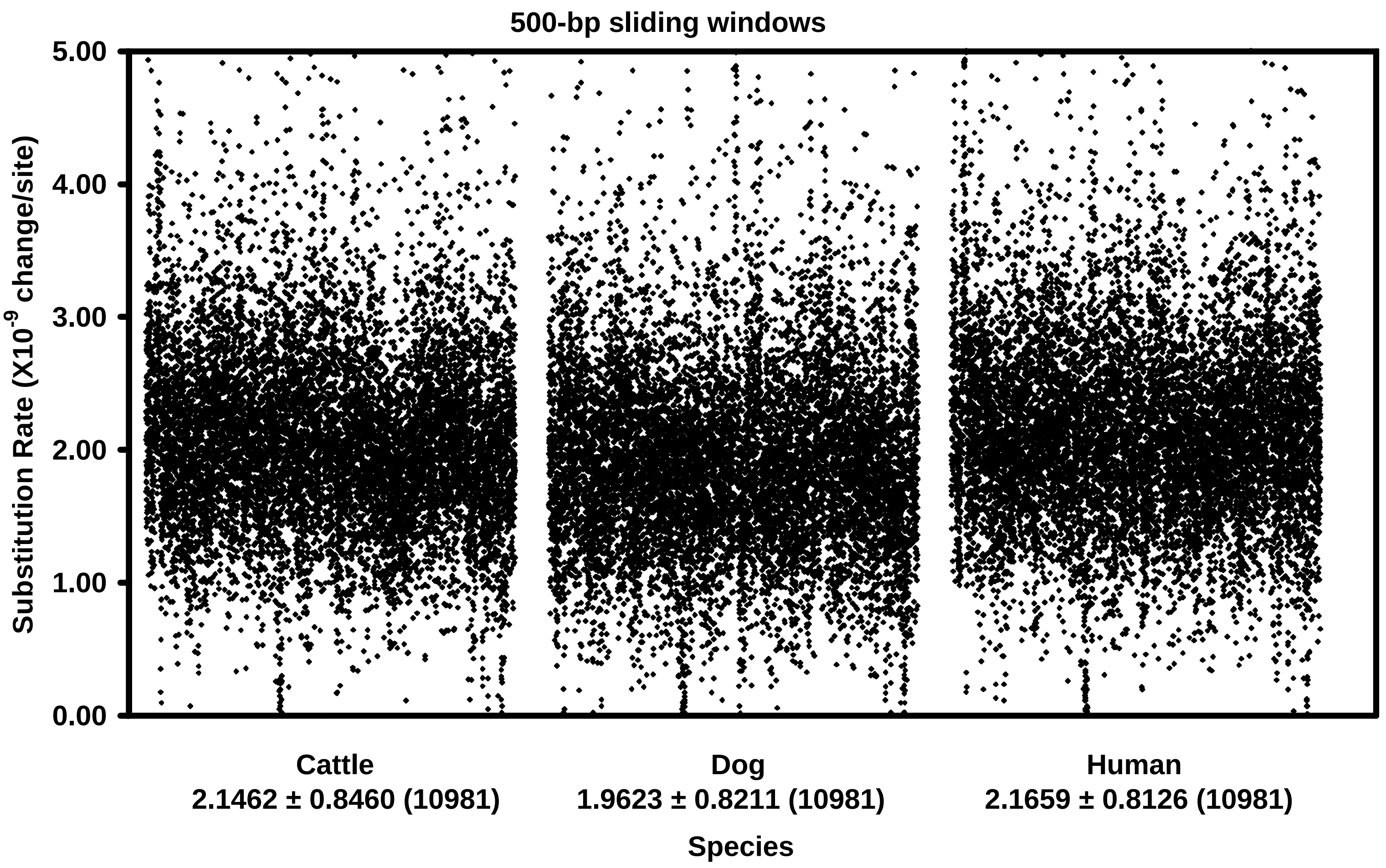
500-bp sliding windows
Substitution Rate (X10^-9 change/site)
5.00
4.00
3.00
2.00
1.00
0.00
Cattle
2.1462 ± 0.8460 (10981)
Dog
1.9623 ± 0.8211 (10981)
Human
2.1659 ± 0.8126 (10981)
Species
